# The Belgian MIRA (MabThera In Rheumatoid Arthritis) registry: clues for the optimization of rituximab treatment strategies

**DOI:** 10.1186/ar3129

**Published:** 2010-09-10

**Authors:** Bert Vander Cruyssen, Patrick Durez, Rene Westhovens, Marie-Joelle Kaiser, Ilse Hoffman, Filip De Keyser

**Affiliations:** 1 Department of Rheumatology, Ghent University Hospital, De Pintelaan 185, 9000 Ghent, Belgium; 2 Department of Rheumatology, Cliniques Universitaires Saint-Luc, Avenue Hippocrate, 10, 1200 Brussels, Belgium; 3 Department of Rheumatology, University Hospitals KULeuven, Herestraat 49, 3000 Leuven, Belgium; 4 Department of Rheumatology, University Hospital Liège, Domaine universitaire B35, 4000 Liège, Belgium; 5 Department of Rheumatology, GZA St-Augustinus Hospital Antwerp, Oosterveldlaan 24, 2610 Wilrijk, Belgium

## Abstract

**Introduction:**

This study describes the results of the Belgian 'MabThera In Rheumatoid Arthritis (MIRA)' registry: effectiveness, safety and evaluation of the current retreatment practice on the background of the Belgian reimbursement criteria for rituximab.

**Methods:**

All Belgian rheumatologists had the possibility to participate in the study. Patients entered the registry in November 2006 and the entry is still open.

**Results:**

By mid-September 2009, 401 patients had entered the registry with a mean follow-up time of 70 weeks. Overall, DAS28-ESR decreased from 6.0 at baseline to 4.2 at week 16. Further decrease of disease activity was observed at the end of year 1 and year 2 with mean DAS28-ESR of 4.0 and 3.7 at these respective time points. More than 80% of patients showed a EULAR response at week 16. Patients could be retreated if they had DAS scores of > 3.2 at least 6 months after the previous course. Second and third courses were given in 224 and 104 patients, respectively. At month 6 after the second course, significantly lower DAS28-ESR values were observed compared to the first course. This was especially the case for patients who were retreated before they showed an obvious flare (DAS increase > 1.2).

**Conclusions:**

This study describes the follow-up of a daily clinical practice cohort of 401 RA patients with long-standing refractory disease treated with rituximab. Relatively high DAS28 values at the start of each retreatment, compared to values 6 months after each treatment course, were noted. Moreover, further decrease of DAS28 scores after the second course was significantly more pronounced in those patients who didn't show an obvious flare. These two elements suggest that treatment of RA patients with rituximab could be optimized by earlier retreatment.

## Introduction

Rituximab (RTX), which has been available for the treatment of lymphoma since 1998, was approved in 2006 for the treatment of rheumatoid arthritis (RA) patients who failed tumor necrosis factor (TNF)-alpha blockers [1]. The need for treatment beyond TNF blockers in RA has become clear since 25% to 40% of patients in clinical trials fail to achieve an ACR-20 (American College of Rheumatology 20% improvement criteria) response [2-4] and a proportion of patients experience treatment-limiting side effects or continue to experience a residual level of disease activity or show flares under anti-TNF therapy.

RTX is a genetically engineered chimeric monoclonal antibody. It binds to the antigen CD20, which regulates cell cycle initiation and differentiation and is found in normal and malignant pre-B and mature B lymphocytes [5,6]. The safety, effectiveness, and prevention of radiological progression by RTX treatment in patients with RA have been proven previously [1,7-9].

The standard course of RTX consists of two 1,000-mg intravenous infusions with an interval of 2 weeks between each dose. Retreatment may be needed between 6 and 12 months after the first course. There is increasing evidence that treatment with repeated courses of RTX over a longer follow-up period is safe and well tolerated [10,11]. However, the retreatment protocol that should be used is still a matter of debate [12].

On the basis of existing evidence about effectiveness, safety, and costs and of approvals by the European Medicines Agency (EMEA) and US Food and Drug Administration (FDA), most countries have developed specific criteria for use of RTX in RA. In Belgium, patients are eligible for RTX treatment if they failed at least one anti-TNF and have a baseline DAS28 (disease activity score using 28 joint counts) of more than 3.7. From week 24, patients may receive further courses of RTX treatment if they had a moderate or good EULAR (European League Against Rheumatism) response at week 16 of the first treatment course and a current DAS28 of at least 3.2. The aims of this study were to evaluate the effectiveness, attrition, and reasons for discontinuation of RTX treatment in daily clinical practice within the reimbursement criteria and to evaluate these criteria.

## Materials and methods

### Study population

The Belgian MIRA (MabThera In Rheumatoid Arthritis) cohort is supported by the Royal Belgian Society for Rheumatology (KBVR/SRBR) via a grant from Roche (Basel, Switzerland). The first patients were recruited in the cohort in November 2006 and recruitment is still open. Recruitment is open to all rheumatologists from Belgium and Luxemburg and covers more than 40% of all academic and non-academic rheumatology centers in those countries.

A specific clinical record file was designed for this study. Baseline variables capture demographics, disease duration, rheumatoid factor and anti-CCP (anti-cyclic citrullinated peptide) status, and (RA) medication history. Additional clinical data are captured at baseline and every 8 weeks from week 16 onwards. These clinical data include the 28 and 66/68 swollen and tender joint counts, erythrocyte sedimentation rate (ESR) (millimeters per hour), C-reactive protein (CRP) (milligrams per liter), patient global visual analog scale (VAS), changes in therapy, and (where applicable) the reason that led to discontinuation; ineffectiveness, safety, elective, and death were predefined in the clinical record file. 'Lost to follow-up' was queried if no data were available at 1 year.

One course of RTX consists of two 1,000-mg infusions RTX with an interval of 2 weeks between each infusion. Prior to the RTX infusion, patients receive 1 g of paracetamol, an antihistaminic (mostly 10 mg cetirizine), and 100 mg methylprednisolon. The study was approved by all ethical committees concerned, and all patients provided written informed consent before recruitment.

### Statistical data analysis

Descriptive analyses were performed by the calculation of means, mean differences (mean diffs), and proportions. Differences between subgroups were analyzed by (paired) *t *tests for continuous variables and chi-square tests for dichotomous variables. Ordinal data were analyzed by the calculation of the gamma statistic. Comparisons between DASs at similar time points from different courses were evaluated by the calculation of differences of means and paired *t *tests. The analysis of different events over time was performed by Kaplan-Meier curves and log-rank tests. Unbalanced data were balanced to an interval of 4 weeks, and missingness completely at random was assumed, and a sensitivity analysis assuming missingness at random was also performed. Statistical analysis was performed with SPSS 17 (SPSS Inc., Chicago, IL, USA). DAS28-ESR values were calculated [13]. A substantial flare was defined as the increase of the DAS28 of at least 1.2 points.

## Results

### Description of the population

By September 2009, data from 401 patients were included in the database with a mean follow-up time of 70 weeks (range 0 to 146 weeks). Patients had a mean age of 59 years (standard deviation 13 years), 76% were female, and the mean disease duration was 12 years (Table 1). The mean DAS28 at baseline was 6.0 (standard error [SE] = 0.1). Second and third courses were given in 224 and 104 patients, respectively.

**Table 1:** Baseline characteristics

Number of patients	401
Age, years	59 (SE 0.6)
Female	76%
Disease duration, years	12 (SE 0.5)
DAS28-ESR at baseline	6.0 (SE 0.1)
RF-positive	81%
Anti-CCP-positive	81%
No previous biologicals	3%
One previous biological	47%
Two previous biologicals	34%
More than two previous biologicals	15%

Anti-CCP, anti-cyclic citrullinated peptide antibody; DAS28, disease activity score using 28 joint counts; ESR, erythrocyte sedimentation rate; RF, rheumatoid factor; SE, standard error.

### Attrition and reasons for discontinuation

Forty-seven patients (12%) discontinued treatment at a median follow-up time of 40 weeks; 30 patients (8%) discontinued because of ineffectiveness and 17 (4%) discontinued because of safety issues. Infusion reactions leading to discontinuation were reported in 6 patients, infections were reported in 4 patients (3 with pneumonia and 1 with recurrent minor infections), and other diverse safety reasons were reported in the remaining 7 patients: the evolution of a pre-existing malignancy in 2 patients (lymphoma and myeloma), leucopenia, mediastinal adenopathies, hair loss, unexplained pain over the body, and hallucinations. One patient died after pneumonia in relation to a hip fracture. Fifteen patients (3.7%) were lost to follow-up.

### Effectiveness

#### Evolution of disease activity for the cohort

Overall, treatment with RTX decreased the cohort's mean disease activity, measured by DAS28-ESR, from 6.0 (SE = 0.1) at baseline to 4.2 (SE = 0.1) at week 16. Eighty-two percent of patients obtained a EULAR response at week 16. Further decrease of disease activity of the cohort was observed at the end of year 1 and year 2 with mean DAS28-ESR values of 4.0 (SE = 0.2) and 3.7 (SE = 0.2), respectively. All four parameters of the DAS28, including the parameters of inflammation, showed a similar trend of decrease over the time: mean ESR values decreased from 38 (SE = 1) to 23 (SE = 2) after 1 year (*P *< 0.001), and mean CRP values decreased from 28 mg/L (SE = 2) to 15 mg/L (SE = 3) (*P *= 0.005).

#### Evolution of disease activity over the different courses

**Figure 1:**
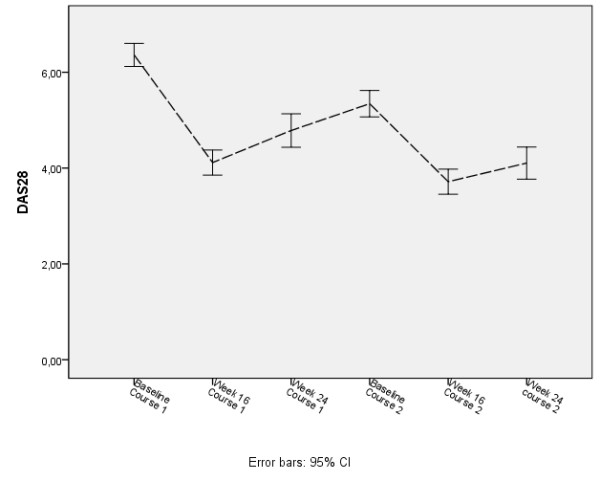
**DAS28-erythrocyte sedimentation rate values at baseline, week 16, and week 24 of each respective treatment course**. Four hundred one patients started rituximab treatment. Second and third courses were given in 224 and 104 patients, respectively. CI, confidence interval; DAS28, disease activity score using 28 joint counts.

At the start of each treatment course, the DAS28-ESR values were lower than values at the start of the previous treatment course. The values reached a minimum at week 16 of each respective treatment course and then increased slightly by week 24 (from week 24 onwards, patients were eligible for retreatment but were not necessarily immediately retreated). From the 24th week, the values further increased until the start of the following treatment course (Figure 1).

**Figure 2:**
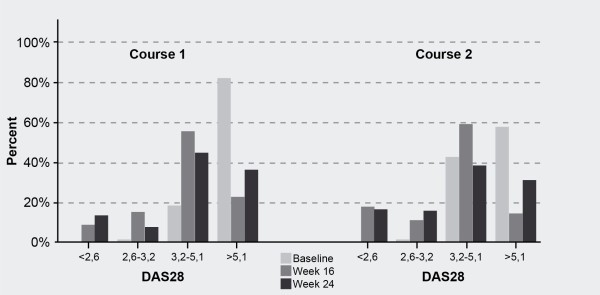
**Proportions of patients in each disease activity score category at baseline, week 16, and week 24 of courses 1 and 2**. Four hundred one patients started rituximab treatment. Second and third courses were given in 224 and 104 patients, respectively. DAS28, disease activity score using 28 joint counts.

**Figure 3:**
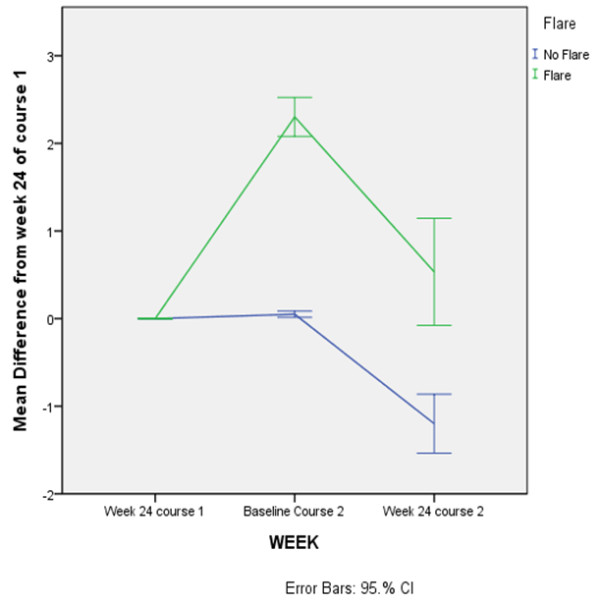
**Evolution of disease activity scores from week 24 on after retreatment in patients with or without flare**. CI, confidence interval.

Paired analysis of DAS28 at 16 and 24 weeks after the first and second courses suggests that lower DASs are obtained after the second course (mean diff at week 16 = 0.04, SE = 0.2, *P *= 0.003; mean diff at week 24 = −0.7, SE = 0.2, *P *< 0.001) (Figure 1). This further decrease of DAS28 between the first and second courses was significantly more pronounced in those patients who did not flare (defined as an increase of DAS28 of greater than 1.2) between week 24 and their second course (mean diff = −1.2, SE = 0.2 versus mean diff 0.2, SE = 0.4, *P *= 0.002). These paired evaluations were performed only in those patients who had received at least two courses and had a follow-up after the second course of at least 24 weeks (Figures 2 and 3).

Similarly, when DAS values were categorized, DAS28-ESR values shifted from higher disease activity segments to lower disease activity segments at each treatment course (Figure 2). At baseline of the first treatment course, 81% of the patients had a high disease activity (DAS of greater than 5.1). This percentage dropped to 22% at 16 weeks, with 23% of patients reaching a remission or low disease activity status (DAS of less than 3.2) at 16 weeks.

At baseline of the second treatment course, 57% of patients had a high disease activity that dropped to 14% at 16 weeks, with 28% reaching a remission or low disease activity status at 16 weeks. Six months after the first and second courses, 80% and 68% of patients, respectively, had at least a moderate disease activity (Figure 2). Second and third courses were given in 224 and 104 patients, respectively.

### Previous use of anti-tumor necrosis factor

**Figure 4:**
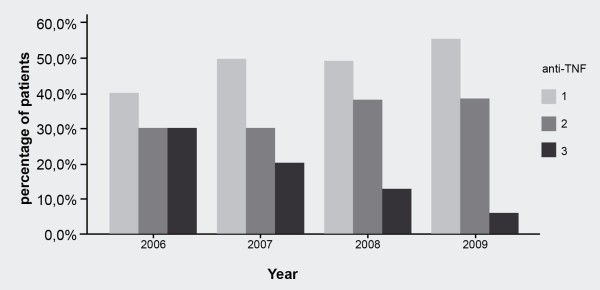
**Number of previously failed anti-tumor necrosis factors (anti-TNFs) in function of time era**.

Data about previous anti-TNF use indicate that the use of RTX is clearly shifting from the use after more anti-TNFs to the use after only one anti-TNF (Figure 4). There was a trend toward lower EULAR response rates at week 16 in patients who failed more than one anti-TNF. Thirty-two percent of patients who failed three anti-TNFs had no response at week 16 in contrast to 13% in patients who failed only one anti-TNF (gamma = −0.22, SE = 0.10, *P *= 0.043) (Table 2).

**Table 2:** EULAR response in function of the number of previously failed anti-TNFs

Number of previously failed anti-TNFs	EULAR response at week 16		
	
	No response	Moderate response	Good response
1 (*n *= 99)	13.1%	59.6%	27.3%
2 (*n *= 72)	18.1%	58.3%	23.6%
3 (*n *= 31)	32.3%	51.6%	16.1%

EULAR, European League Against Rheumatism; TNF, tumor necrosis factor.

## Discussion

This study describes the follow-up of a cohort of 401 RA patients with longstanding refractory disease treated with RTX in combination with methotrexate. These patients are treated and followed in a daily clinical practice setting at different rheumatology centers in Belgium or Luxemburg. The high coverage of this study by the different rheumatologists and the low percentage of 'lost to follow-up' (3.7%) suggest a good representation of the patients who are candidates for this treatment.

At week 16, 82% of patients obtained a good or moderate EULAR response, with 23% of patients reaching a remission or low disease activity status after only one treatment course and 28% to 32% (week 16 and week 24) after the second treatment course. This suggests that the effectiveness of RTX after a first course is similar in this daily clinical practice setting to the effectiveness seen in more controlled (open-label and placebo-controlled) trials and studies [1,8].

Moreover, even lower DAS values can be obtained after a second course of RTX: paired analysis of DASs at week 16 and week 24 from the first and second courses showed significantly lower DASs after the second course of RTX. This is especially the case for patients who were retreated before they flared. A further decrease of disease activity after the second course has been previously suggested [10], but limited data are available on the effect of retreatment before flare. One controlled trial suggested no difference in response between fixed versus on-demand retreatment strategies [12]. However, recently presented retrospective data [14] and unpublished data from a randomized controlled trial [15] suggest a clear difference between the efficacies of the two approaches. Thurlings and colleagues [16] demonstrated that, when a systematic retreatment approach was applied, a second RTX course in first-course responders resulted in a further decrease of DAS28 of 1.2 points.

The data from the present study suggest that lower cumulative DAS values can be obtained if patients with a DAS28 of more than 3.2 are retreated before they show a substantial flare. The further DAS28 decrease in these patients (−1.2) is comparable to the DAS28 decrease reported by Thurlings and colleagues [16] when a systematic retreatment approach was applied. Overall, systematic retreatment if patients show a DAS28 of more than 3.2 is in line with the concept of DAS-driven tight control of the disease; at the moment, this seems to be the best strategy to induce persistent low disease activity, at least in patients with early RA [17]. Therefore, the possibility of a systematic retreatment after 6 months in patients with a residual disease activity of more than 3.2 is an important aspect of the current reimbursement criteria.

It has been suggested that the number of failed anti-TNFs and the serological status are predictors of response to RTX therapy [18]. In this study, we confirmed that the number of previously failed anti-TNFs was significantly associated with response to the first course of RTX: 32% of patients who failed three anti-TNFs had no response at week 16 in contrast to 13% in patients who failed only one anti-TNF. Previous reports suggested that the switch to RTX after failing one anti-TNF may be more effective than switching to an alternative anti-TNF agent in patients with an inadequate response to anti-TNFs [19,20]. These data, together with the growing experience of clinicians, may explain the observation that the proportions of patients who failed three anti-TNFs before starting RTX have been decreasing over the years in favor of a switch after failure of one or two anti-TNFs.

In line with previous data [10,21], RTX appears to be safe: 17 (4.3%) patients discontinued because of safety issues (the most prevalent of which was allergic infusion reaction). Infections were reported in 4 patients (3 with pneumonia and 1 with recurrent minor infections). One patient died following pneumonia in relation to a hip fracture. No cases of progressive multiple leukoencephalopathy were seen in this cohort [22].

This study has strengths and weaknesses. Similar to all observational studies, this study may suffer from expectation bias, leading to an overestimation of the response rates to RTX therapy. Additionally, there was no protocol guiding the decision for retreating patients (and this has led to heterogeneity between centers and patients) or the evolutions of treatment strategies over time. It is especially important to notice that, following the Belgian reimbursement criteria in the present study, patients could be retreated only if they showed a remaining disease activity of greater than 3.2. Therefore, the potential benefit of systematic retreatment could be evaluated only in patients with at least moderate remaining disease activity. The strength of this study is that the registry was open to all Belgian and Luxembourgian rheumatologists, resulting in a high participation rate of small and large rheumatology centers. Consequently, this study gives a representative view of the daily clinical practice use of RTX.

## Conclusions

From this daily clinical practice cohort, some recommendations for the optimization of RTX treatment strategies can be given: If patients can be retreated before they show an obvious flare, a more pronounced further decrease of disease activity can be observed. Therefore, systematic retreatment of patients with a remaining disease activity of DAS greater than 3.2 should be considered. Also, as DAS values may further decrease after the second course, a switch to another treatment strategy for unacceptable remaining disease activity should be considered at the earliest after the second course.

## Abbreviations

CRP: C-reactive protein; DAS: disease activity score; DAS28: disease activity score using 28 joint counts; ESR: erythrocyte sedimentation rate; EULAR: European League Against Rheumatism; mean diff: mean difference; RA: rheumatoid arthritis; RTX: rituximab; SE: standard error; TNF: tumor necrosis factor.

## Competing interests

This study is supported by the Royal Belgian Society for Rheumatology (KBVR/SRBR) via a grant from Roche. BVC is a post-doctoral researcher supported by the FWO Flanders and received speakers fees from Roche, Wyeth (Madison, NJ, USA), Schering-Plough Corporation (Kenilworth, NJ, USA), and Abbott (Abbott Park, IL, USA). PD received speakers fees from BMS (New York, NY, USA), Wyeth, and Schering-Plough Corporation. RW is a consultant for Roche, BMS, and Centocor, Inc. (Horsham, PA, USA) and received a research grant from UCB (Brussels, Belgium). IH received speakers fees from Roche, Abbott, Schering-Plough Corporation, and BMS. FDK has received research grants from Roche. M-JK declares that she has no competing interests.

## Authors' contributions

BVC participated in the study design, performed the statistical analysis, constructed the datasets, and drafted the manuscript. RW, FDK and PD participated in the study design. All authors contributed to the writing of the article and read and approved the final manuscript. All doctors from the MIRA Study group recruited and followed the arthritis patients.
